# Prediction of Deterministic All-Optical Switching of Ferromagnetic Thin Film by Ultrafast Optothermal and Optomagnetic Couplings

**DOI:** 10.1038/s41598-017-13568-w

**Published:** 2017-10-18

**Authors:** Zhidong Du, Chen Chen, Feng Cheng, Yongmin Liu, Liang Pan

**Affiliations:** 1 School of Mechanical Engineering and Birck Nanotechnology Center, Purdue University, West Lafayette, Indiana, 47907 USA; 2 Department of Mechanical and Industrial Engineering, Northeastern University, Boston, Massachusetts, 02115 USA

## Abstract

All-optical switching (AOS) of magnetization induced by ultrafast laser pulses is fundamentally interesting and promises unprecedented speed for magnetic data storage that is three orders of magnitudes faster than the current techniques. For ferrimagnetic material, the AOS is attributed to magnetic circular dichroism and angular momentum transfer between sublattices. Recently, ferromagnetic material is demonstrated in AOS under multiple pulses. Since the magnetic field needed to flip the ferromagnetic magnetization within femtosecond timescale is unphysically high, some theories hypothesized that there exists a prolonged magnetic field beyond the pulse duration in the switching process. This is intuitively inconsistent with the phenomenological explanation based on the light-induced magnetic field arising from the inverse Faraday effect (IFE). Here, we numerically study the AOS process and provide new insights into the long-standing paradox of the duration of the induced magnetic field. We show that the prolonged magnetic field duration originates from the ultrafast optothermal and optomagnetic coupling. Moreover, we numerically studied both single- and multiple-pulse AOS under different coupling strength between spins and the thermal bath in the macroscopic Fockker-Planck and Landau-Lifshitz-Bloch model. This numerical model may provide a guide to find suitable ferromagnetic materials for AOS.

## Introduction

Magnetization and spin manipulation can be achieved by electric fields^1–4^, spin-polarized currents^5–11^ and ultrafast laser pulse^12–15^, without applying an external magnetic field. In particular, ultrafast optical manipulation of magnetization has emerged as an exciting direction in modern magnetism, since the discovery of ultrafast demagnetization of a nickel film by a 60-femtosecond laser pulse^12^. Subsequent work not only confirmed the effect^16–19^, but also demonstrated the possibility to optically generate coherent magnetic precession^20,21^ and optically induce spin reorientation^22^. One of the widely-discussed topics in ultrafast magnetization manipulation is that circularly polarized laser pulses can directly and deterministically switch magnetic domains without applying external magnetic field^23–25^. This is termed as all-optical helicity dependent switching or simply as all-optical switching (AOS)^13,26^. While early AOS studies were focused on ferrimagnetic GdFeCo alloys, subsequent studies have expanded to other rare earth-transition metal materials, synthetic ferrimagnets, and very recently to ferromagnetic materials such as CoPt and other magnetic thin films, multilayers and granular films^23,25,27–31^.

Despite the successful demonstrations of AOS, the role of ultrafast laser pulses in AOS is still under debate. Experimental and theoretical studies suggest that several mechanisms may be important in the determinacy of flipping, which include the inverse Faraday effect (IFE)^24,32–34^, magnetic circular dichroism^28,35–37^, the transfer of angular momentum^38–40^, the formation of a transient ferromagnetic state^35^ and laser-induced superdiffusive spin currents^10,40,41^. For the ferrimagnetic material, AOS may be explained as a result of angular momentum transfer between the two ferrimagnetic sublattices^28,34^. But for the ferromagnetic material recently demonstrated, this explanation is not applicable due to the lack of sublattices. When the AOS first came out, it was generally believed that circularly polarized laser pulses have two-fold effects in the AOS process. First, it energizes the electrons in the magnetic material and the demagnetization happens because the electron temperature increases above the Curie temperature within about picosecond timescale^42^. Second, a magnetic field is induced by the circularly polarized laser to achieve switching during the settling process of the energized magnetic material. Because the origin of the inverse Faraday effect in the AOS system is still unclear, there is no direct measurement of the amplitude or the duration of the induced magnetic field. Several attempts have been made to explain the flipping process using phenomenological assumptions to prolong the induced magnetic field duration^13,23,24,34^. However, such assumptions are seemingly inconsistent with the fact that inverse Faraday effect should disappear together with the laser pulse. Here we numerically studied the AOS process and confirmed the existence of the prolonged induced magnetic field. We showed that the prolonged induced magnet field originates from the complicated optothermal and optomagnetic couplings at femto- and pico-second timescales, which are not directly observable in experiments. Moreover, we derived a simplified analytical model to predict the AOS which can be used by other researchers without the needs of performing intense computations. The simulation results show that the single laser pulse AOS in ferromagnetic system is possible under some optical, thermal and magnetic parameters, but it hasn’t been demonstrated experimentally. Using this model, the observed ferromagnetic AOS under multiple pulses and the edge-dominated flip are also reproduced to show the feasibility of this model. This work will allow prediction of parameter regions where the AOS process could happen and help development of ultrafast magnetic data recording and nanomagnetic devices.

## Results

**Figure 1:**
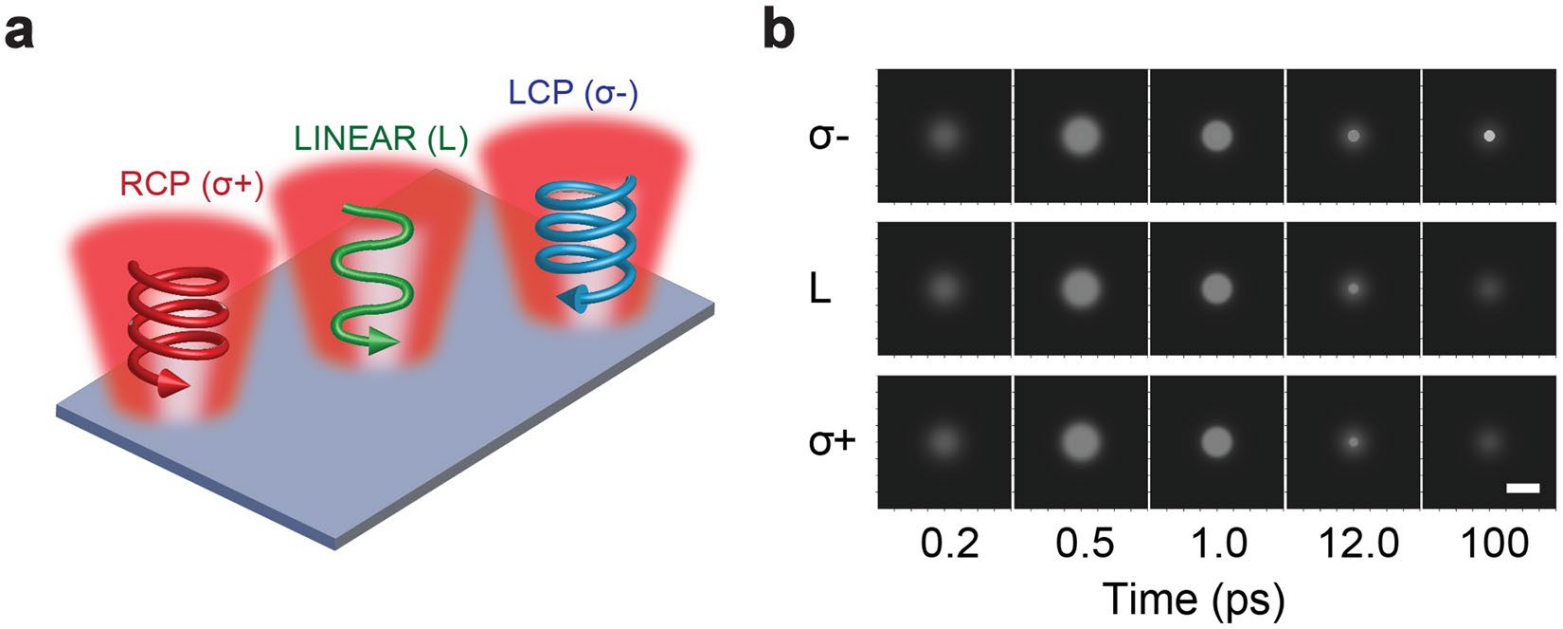
All-Optical Switching (AOS) scheme and time evolution of magnetization. (**a**) Illustration of AOS. Laser pulses heat up the magnetic material, and the induced magnetic field arising from inverse Faraday effect (IFE), which can either flip or preserve the final magnetization depending on the helicity of laser pulses. (**b**) The time evolution of magnetization subjected to a single pulse of left-handed (σ^−^), linearly (L) and right-handed (σ^+^) polarized light. Black and white areas correspond to up (M^+^) and down (M^−^) magnetic domains. The laser fluence is 2.6 mJ/cm^2^ with 140 fs duration (full width at half maximum) and the beam diameter (defined by 1/*e* of peak intensity) is 10 μm. The magneto-optical susceptibility *α* is set to be 2.13 × 10^−11^ A∙m/V^2^ (corresponding to 1 T effective field produced by 1 mJ/cm^2^ laser fluence). The scale bar is 10 μm.

In our study, we use a two-temperature model to capture the thermal response of electron and lattice systems^43–45^. A macroscopic Fockker-Planck and Landau-Lifshitz-Bloch (LLB) model is used to capture the magnetization dynamics of a ferromagnetic thin film under the pulsed laser irradiation^46–51^. Figure 1a schematically illustrates AOS, along with the definitions of three different helicities for right-circularly polarized (RCP or σ^+^), linearly polarized (L) and left-circularly polarized (LCP or σ^−^). The LCP and RCP pulses induce effective magnetic fields in opposite directions, through the inverse Faraday effect^52–55^, where a circularly polarized light can produce an effective magnetic field. More details about multiphysics numerical implementations can be found in Method section.

Figure 1b presents three representative cases of AOS processes reproduced in this numerical study under laser pulses with different helicities. Domains with magnetization up (M^+^) and down (M^−^) show black and white contrast, respectively. The initial magnetic domain has a uniform magnetization up state. In our simulations, the wavelength of the laser is 800 nm, and the laser pulse has a 140-fs duration, 2.6 mJ/cm^2^ fluence and a beam diameter of 10 μm. The virtual magnetic material is chosen to be ferromagnetic with a thickness of 10 nm without material inhomogeneity and the substrate is fused silica (see detailed numerical implementations in the Method section). These parameters are chosen based on previous experimental and theoretical work^15,30,34,56^. After the irritation of a single pulse with different helicities, the magnetization evolutions are shown at 0.2, 0.5, 1.0, 12.0 and 100 ps, respectively. One can clearly see that under the 2.6 mJ/cm laser fluence, the magnetization up (M^+^) domain can be flipped by the laser pulse with opposite helicity (σ^−^) while the linear and RCP laser pulses cannot flip the M^+^ domain. These results show that the single pulse AOS for ferromagnetic magnetic material is possible though it hasn’t been confirmed experimentally. If the single pulse ferromagnetic AOS existed, it would show similar time evolution as the ferrimagnetic material^23,24^.

### Time evolution of the AOS magnetization

**Figure 2:**
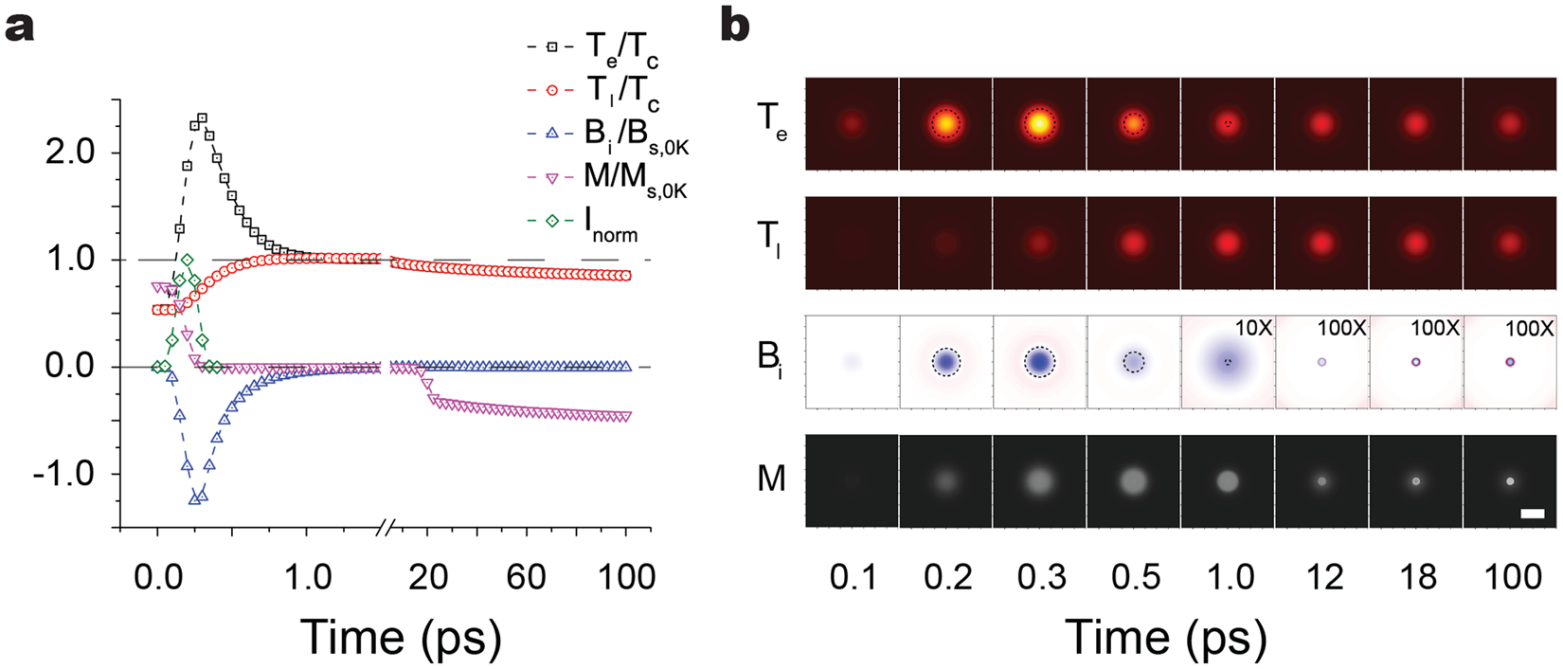
Time evolution of the four important quantities during the deterministic switching: electron temperature (*T*_*e*_), lattice temperature (*T*_*l*_), induced magnetic flux density *B*_*i*_ and magnetization *M*, after a single LCP (σ^−^) pulse irradiates onto an M^+^ magnetic medium. (**a**) Time evolution of these quantities at the center of the laser beam. *T*_*e*_ and *T*_*l*_ are normalized to Curie temperature *T*_*C*_. The *M* and *B*_*i*_ fields are normalized to *M*_*s,0K*_ and *B*_*s,0K*_ (or *µ*_*0*_ *M*_*s,0K*_) respectively. Here *M*_*s,0K*_ is the saturated magnetization at zero temperature. The *I*_*norm*_ represents the normalized laser pulse. (**b**) The maps of *T*_*e*_, *T*_*l*_, *B*_*i*_ and *M* in the middle plane of the 10 nm thick ferromagnetic film. The *B*_*i*_ field is magnified by 10 times at 1.0 ps and 100 times at 12.0, 18.0 and 100 ps. The dashed circles in the *T*_*e*_ and *B*_*i*_ maps enclose the areas where *T*_*e*_ is larger than *T*_*C*_. The scale bar is 10 μm.

Figure 2 shows the time evolutions of four key physical quantities at the beam center, including electron temperature (*T*_*e*_), lattice temperature (*T*_*l*_), induced magnetic flux density (*B*_*i*_) and magnetization (*M*), during the process of deterministic AOS driven by an LCP (σ^−^) pulse. The parameters used here are the same as Fig. 1b. During the first a few hundreds of femtoseconds, the laser pulse irradiates the magnetic film and induces optomagnetic coupling through the inverse Faraday effect and wakes a loop current field, which both contribute majorly to the overall induced B field (*B*_*i*_). At the rising edge of the laser pulse, the induced B field ramps up together with the increasing *T*_*e*_ and *H*_*IFE*_. Meanwhile, the change of the magnetic flux (*Φ*_*B*_) induces a vortex electric field E which displaces the electrons to form a loop current ***j*** which also contributes to the overall induce B field (*B*_*i*_). According to Lenz’s law, the direction of the B field created by the loop current ***j*** counteracts with the changing magnetic field, which causes a time delay of about 60 fs between the peaks of the laser pulse and the overall induced B field. After *H*_*IFE*_ vanishes together with the laser pulse, the induced B field still exists because the loop current ***j*** sustained by the decreasing magnetic flux *Φ*_*B*_. As one can see from the blue curve B_i_/B_s,0K_ in Fig. 2a, the induced magnetic field decays to 1/*e* of its peak value at about 460 fs, which lasts significantly longer than the laser pulse duration. The distribution of *B*_*i*_ is shown in Fig. 2b at different times. It is magnified by 10 times at 1 ps and 100 times at 12, 18 and 100 ps to be seen more clearly. Evidence of this prolonged induced B field was observed in the pump-probe experiments of the magnetization evolution where the flipping was shown to occur at picosecond timescale^23,24,57^.

In the meantime, the laser pulse brings the magnetic medium into a strong non-equilibrium state through the optothermal coupling. Because of the small heat capacity of electron system, the electron temperature rapidly increases (black curve *T*_*e*_/*T*_*C*_) and peaks to 2.33 times of the material’s Curie temperature (*T*_*C*_ = 550 K) within 275 fs. This elevated electron temperature drags the lattice temperature (red curve *T*_*l*_/*T*_*C*_) through the electron-lattice coupling to increase gradually at a much slower rate. During this time period, the original magnetization (M^+^) is nearly quenched (magenta curve *M*/*M*_*s*,0*K*_, where *M*_*s*,0*K*_ is the saturation magnetization at 0 K and has the value of 8 × 10^5^ A/m), and the ferromagnetic material changes into its paramagnetic phase. At this sub-picosecond timescale, other physical effects (such as domain walls and magnetic precession) can be numerically treated as magnetic disturbances because they do not reproducibly or strongly affect the evolutions of these key system quantities due to their temporal and spatial characteristics.

After the laser pulse, the electron and lattice systems gradually approach their local equilibrium and the temperature of the magnetic film drops due to thermal diffusion. As the material temperature crosses the Curie temperature, its paramagnetic phase relaxes back to its original ferromagnetic phase. During this phase restoration, the remaining of the induced B field is still sufficiently strong to outbalance the thermal or surrounding disturbance and preferably select a path at the beginning of the remagnetization process. Due to the dynamic nature for ferromagnetism, this initial selection self-reinforces as time elapses. After 20 ps, the temperature drops further below the Curie temperature and the magnetic thin film is deterministically flipped. As for excitations by linearly (L) or RCP (σ^+^) pulses with the same fluence and beam size, the magnetic material undergoes a demagnetization process and eventually recovers to its original magnetization (Fig. S1 in Supplementary materials).

Depending on the initial magnetization map, the ultrafast laser pulse can also cause temporal and spatial variations of M field in the irradiated area, therefore induce a weak B field near the center of the laser beam. When the film has a uniform initial magnetization, the variation of M field between the demagnetized center and the unaffected surrounding domains can induce a B field with a similar shape to that of a ring magnet and in the opposite direction of the surrounding M field and irrelevant to the laser helicities. Because of the nanoscale film thickness, this B field induced by M field variations is negligible at the early stage when compared with other effects, but may become important when other effects are absent or cancelled out.

### Threshold of the laser pulse fluence for AOS

**Figure 3:**
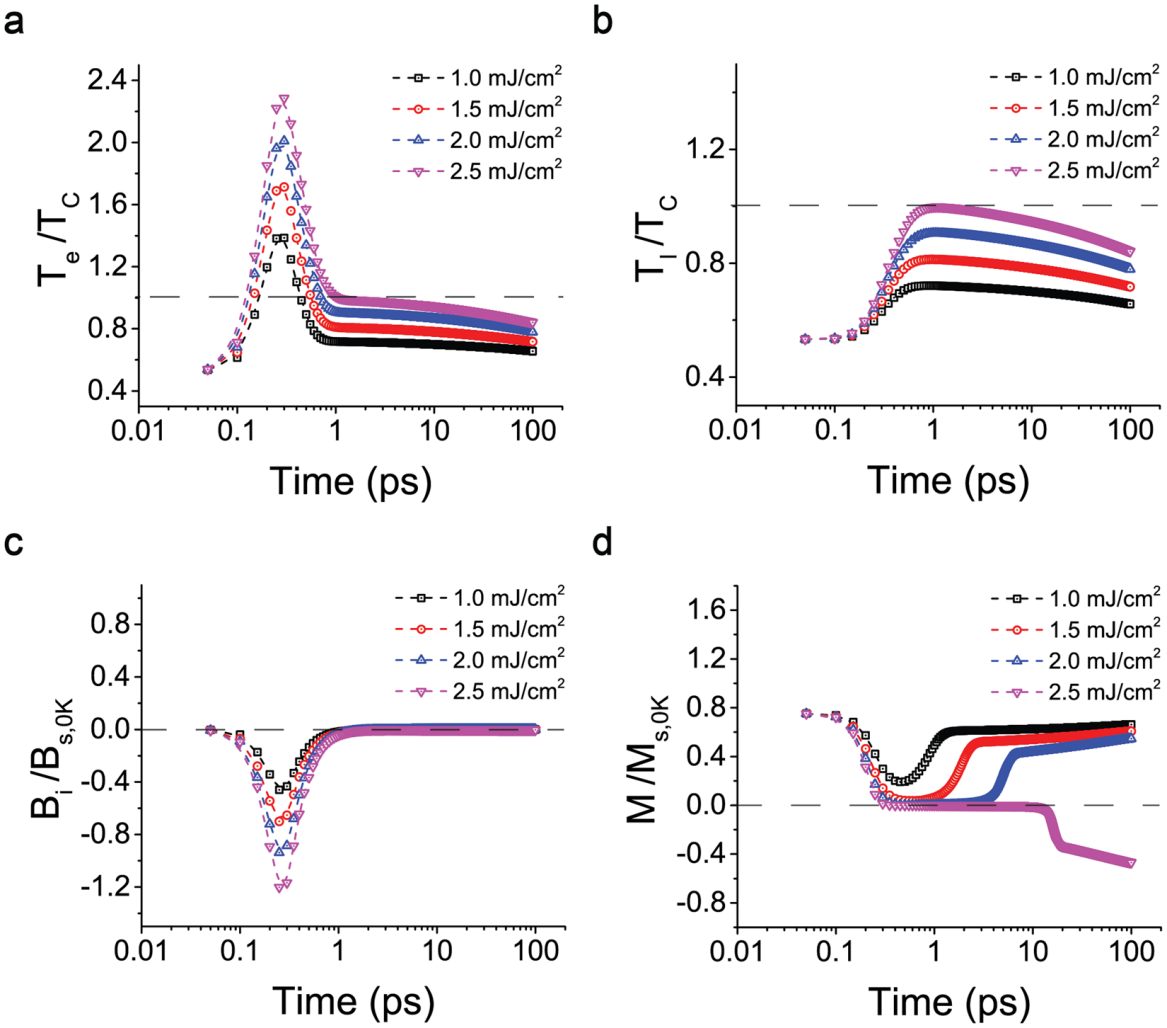
Switching threshold of the laser fluence. Time evolutions of *T*_*e*_, *T*_*l*_, *B*_*i*_ and *M* under different laser fluences, where switching occurs at about 2.5 mJ/cm^2^. The laser beam diameter is 10 μm.

It has been experimentally and numerically demonstrated that the laser fluence is a critical parameter in AOS^23,24,30,34^. For ferrimagnetic material, the laser fluence threshold is determined by the magnetic circular dichroism that the circular light is absorbed differently by different magnetizations^28^. For ferromagnetic material AOS, the threshold comes from that the demagnetization is required for the induced magnetic field to flip the spin. Figure 3a–d shows the material responses under different laser fluences, while the other parameters are the same as those in Fig. 2. The laser fluence directly drives two mechanisms for AOS, which are induced B field and thermal demagnetization. The induced B field increases monotonically with respect to the laser fluence. And the demagnetization process is predominately driven by the thermal energy carried by the ultrafast laser pulse. At the laser fluence of 2.5 mJ/cm^2^ or not far above (magenta curves for 2.5 mJ/cm^2^ fluence), the laser pulse can excite the electron temperature well above its Curie temperature (reaches about 2.3 T_C_) within 1 ps which preferably quenches the original magnetization and triggers AOS to occur. As laser fluence reduces to 2.0 mJ/cm^2^ shown in blue curves (1.5 mJ/cm^2^ in red and 1.0 mJ/cm^2^ in black), the pulse energy can still pull the material towards zero magnetization (*M/M*_*s*,0*K*_) reaching 0.0028 (0.038 and 0.19) at 0.65 ps (0.55 ps and 0.45 ps). However, at the time when this minimum magnetization is achieved, the electron temperature has already dropped below Curie temperature and therefore the strength of prolonged magnetic field becomes insufficient to prevent the material restoring to its original magnetization, therefore no AOS occur. At the fluence higher than 2.8 mJ/cm^2^, the thin film will undergo demagnetization where the final magnetization state is no longer determined by the laser pulse helicity (Fig. S2 in Supplementary materials).

### Characteristics of the induced magnetic field

**Figure 4:**
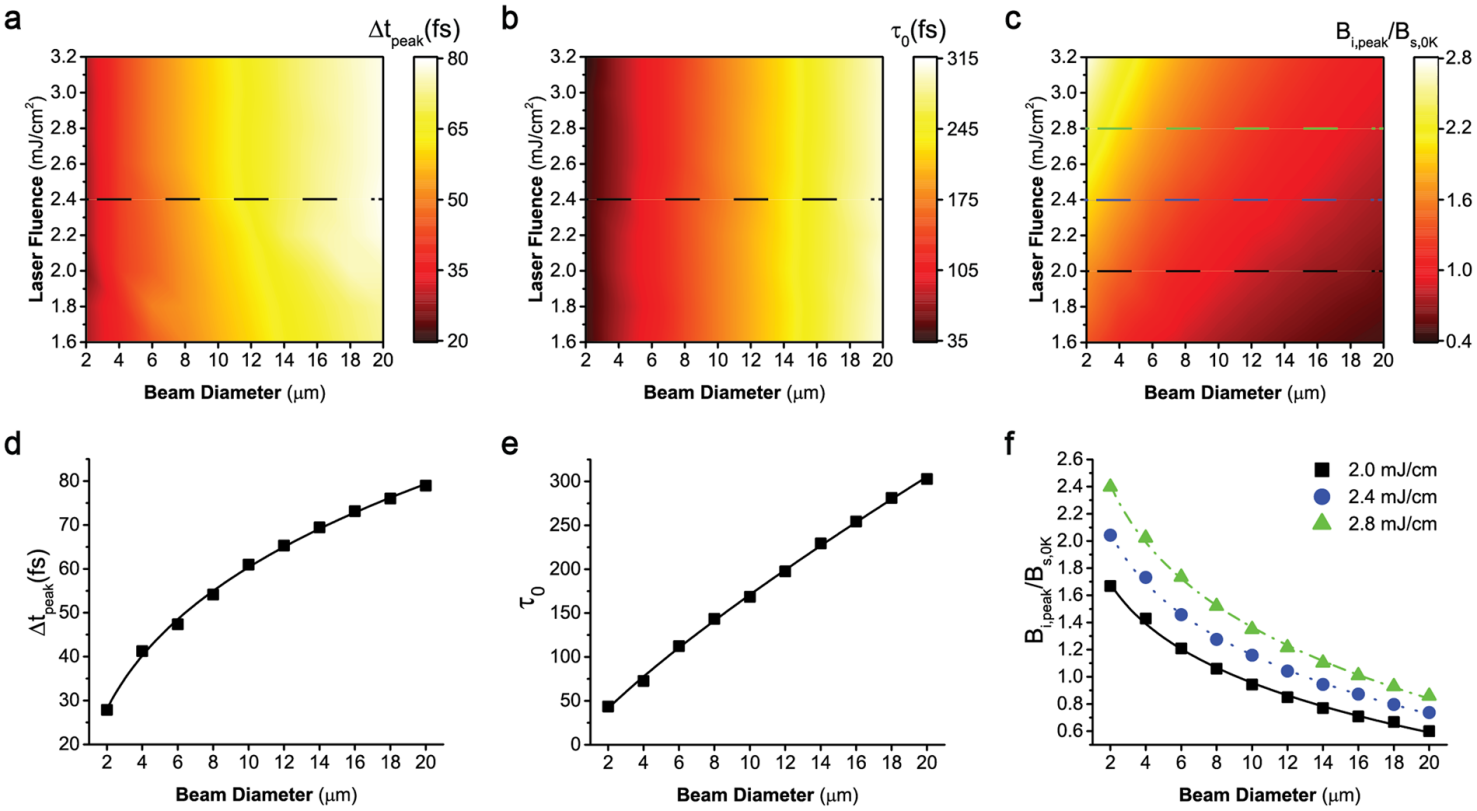
Dependence of the parameters of the induced magnetic field *B*_*i*_ on the laser fluence and beam diameter for (**a**) the postponed time ∆*t*_*peak*_ between the peaks of the laser pulse and *B*_*i*_, (**b**) the decay constant time *τ*_*0*_ of *B*_*i*_, and (**c**) the peak value of *B*_*i,peak*_/*B*_*s,0K*_. (**d–f**) The value of ∆*t*_*peak*_, *τ*_*0*_ and *B*_*i,peak*_/*B*_*s,0K*_ along the dashed lines. The *B*_*i*_ field is calculated at the center of the laser beam.

The induced B field at the beam center can be estimated by the convolution of a magnetic pulse excitation *f*(*t*) caused by the inverse Faraday effect and a relaxation function *g*(*t*) caused by the loop current if the impact of the *M* field can be neglected. The system relaxation response under a δ stimulus can be approximated as an exponential decay. So the estimated *B*_*i*_ can be written as *B*_*i =*_ *B*_0 +_ *f*(*t*)⊗*g*(*t*), where *B*_0_ is the background B field. There are three important parameters: the postponed time (∆*t*_*peak*_) of the peak position with respect to the peak of the laser pulse, the peak magnitude (*B*_*i,peak*_), and the effective decay time constant (*τ*_0_) for the relaxation function *g*(*t*). A larger value in any of these three parameters would favor AOS to occur by producing a sufficiently large *B*_*i*_ field particularly during the time window when the original *M* field is almost quenched. Figure 4a–f shows the impacts on these three parameters at the center of the laser beam from the beam diameter (2-20 μm) and the laser fluence (1.6–3.2 mJ/cm^2^). When the laser diameter increases, the response of the induced loop current density slows down due to the increasing effective inductance. As a consequence, the enlarged beam size will help to increase the postponed time ∆*t*_*peak*_ (Fig. 4a) and the decay time constant *τ*_0_ (Fig. 4b), however, reduce the peak of the induced magnetic field *B*_*i,peak*_ (Fig. 4c). In contrast, changing the laser fluence would only play a strong role in *B*_*i,peak*_ (Fig. 4c) leaving ∆*t*_*peak*_ (Fig. 4a) and *τ*_0_ (Fig. 4b) nearly unaffected. This is because the laser pulse fluence can directly drive *B*_*i,peak*_ through inverse Faraday effect, but only influences ∆*t*_*peak*_ and *τ*_0_ through other weak effects such as thermally inducing the *M* field variations. When the thermally induced *M* field variation is negligible, the *B*_*i*_ field reduces to an explicit function of the laser fluence only and ∆*t*_*peak*_ and *τ*_0_ appear nearly independent of the laser fluence. These obtained decay time constants in Fig. 4 agree well with the trends in the phenomenological models used in previous work^13,23,24,34^.

### Phase diagrams of AOS

**Figure 5:**
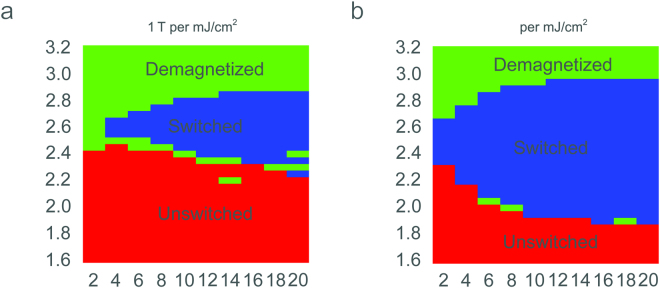
Phase diagrams of AOS with different magneto-optical susceptibilities. The magneto-optical susceptibilities correspond to (**a**) 1 T per mJ/cm^2^ and (**b**) 10 T per mJ/cm^2^ respectively. The AOS thresholds (boundaries between the switched and unswitched domains) are determined by the threshold of flipping under σ^-^ polarized laser pulse. The demagnetization thresholds (boundaries between the switched and demagnetized domains) are determined by the thresholds of flipping under σ^+^ polarized laser pulse. The initial magnetic state is M^+^.

The thresholds of AOS is influenced by several parameters such as laser beam diameter, laser fluence, and magneto-optical susceptibility. Figure 5a,b shows the results of the phase diagrams of deterministic AOS at the center of the laser beam, under two different magneto-optical susceptibilities corresponding to 1 T and 10 T per mJ/cm^2^ for the left and right panels respectively. The unswitched, deterministically switched, and thermally demagnetized regions are labelled in red, blue, and green respectively. The equivalent level of the magnetic noise caused by numerical errors is about a few Gauss at the beam center. In the unswitched region, the material can recover to its original magnetization disregard the helicities of laser pulses. The height of the deterministically switched region increases with both beam size and inverse Faraday effect. For the 1 T per mJ/cm^2^ case, no AOS occurs at the 2-μm beam size for the material used here mainly due to the low peak and fast decay of *B*_*i*_ field. The regions are considered demagnetized where their final states are not solely determined by their original states or the helicities of laser pulses. As for the demagnetization threshold, because the thermal or magnetic disturbance is not included in our multi-physics model, we use the threshold of magnetic flipping under the same laser helicity as the initial *M* field. The reason to use this threshold is that in the portion above the switched region, the temperature stays above the Curie point for a long time and outlives the induced *B*_*i*_, so even under σ^+^ polarized laser pulses the M^+^ field can still be flipped to M^−^ and the final magnetic states are mainly determined by neighboring magnetic environments. A narrow band of transition region also exists to continuously separate the unswitched and deterministically switched region (appears discontinuous in the figure mainly due to discretized grids and numerical noise). In this lower transition band regions, the overall effect of the induced *B*_*i*_ is balanced by other counteracting effects leading to random remagnetizations.

**Figure 6:**
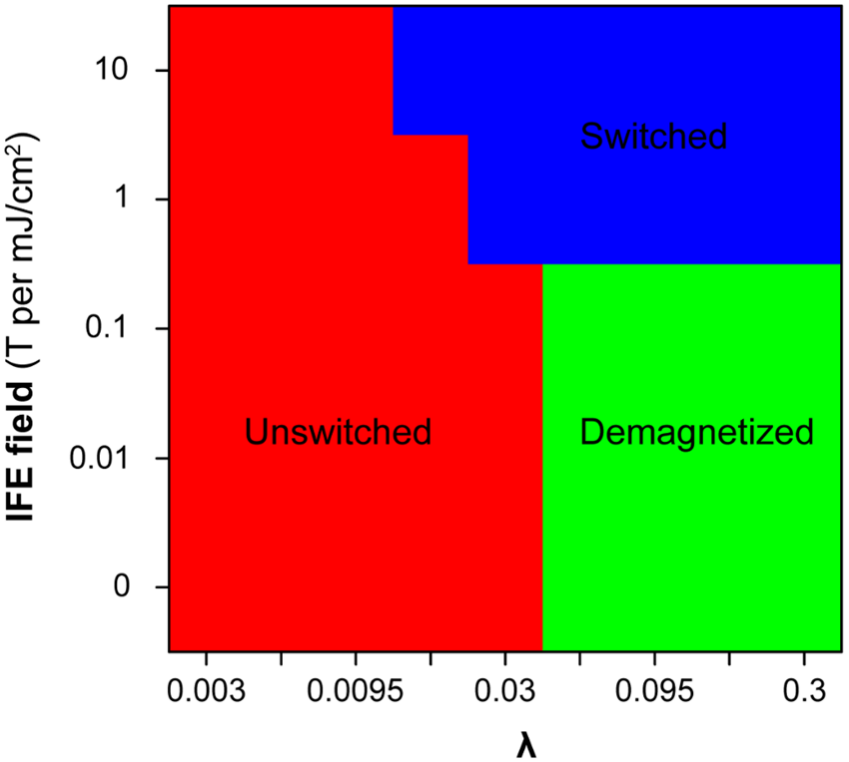
Phase diagrams of AOS with different IFE field and thermal coupling strength λ between the spin and the thermal bath. The laser fluence is kept at 2.6 mJ/cm^2^ with 10 μm diameter laser beam. The IFE field is changed from 0 to 10 T per mJ/cm^2^. The thermal coupling strength λ between the spin and the thermal bath is changed from 0.003 to 0.3. For one finite temperature, the Gilbert damping rate is proportional to the coupling strength λ shown in supplemental material.

The impact of the temporal response of the virtual ferromagnetic material is studied by varying the thermal coupling strength λ between the spin and the thermal bath. Figure 6 studies the phase diagrams of AOS with different IFE field and thermal coupling strength λ between the spin and the thermal bath. The laser fluence is kept at 2.6 mJ/cm^2^ with 10 μm diameter laser beam. The IFE field is changed from 0 to 10 T per mJ/cm^2^. The thermal coupling strength λ between the spin and the thermal bath is changed from 0.003 to 0.3. For one finite temperature, the coupling strength λ is proportional to the Gilbert damping rate shown in supplemental material. And the larger the coupling strength is, the faster the ferromagnetic material responds. It is shown that the ferromagnetic material needs to respond fast enough to allow AOS happening within the time window of the prolonged induced magnetic field. The IFE field also needs to be large enough to deterministically flip the spins. These two requirements explain the switched region shown in blue color. If the ferromagnetic material responds fast but the IFE field is not large enough, the quenched magnetization will be guided by the ambient magnetic field. This is the demagnetized region shown in green.

**Figure 7:**
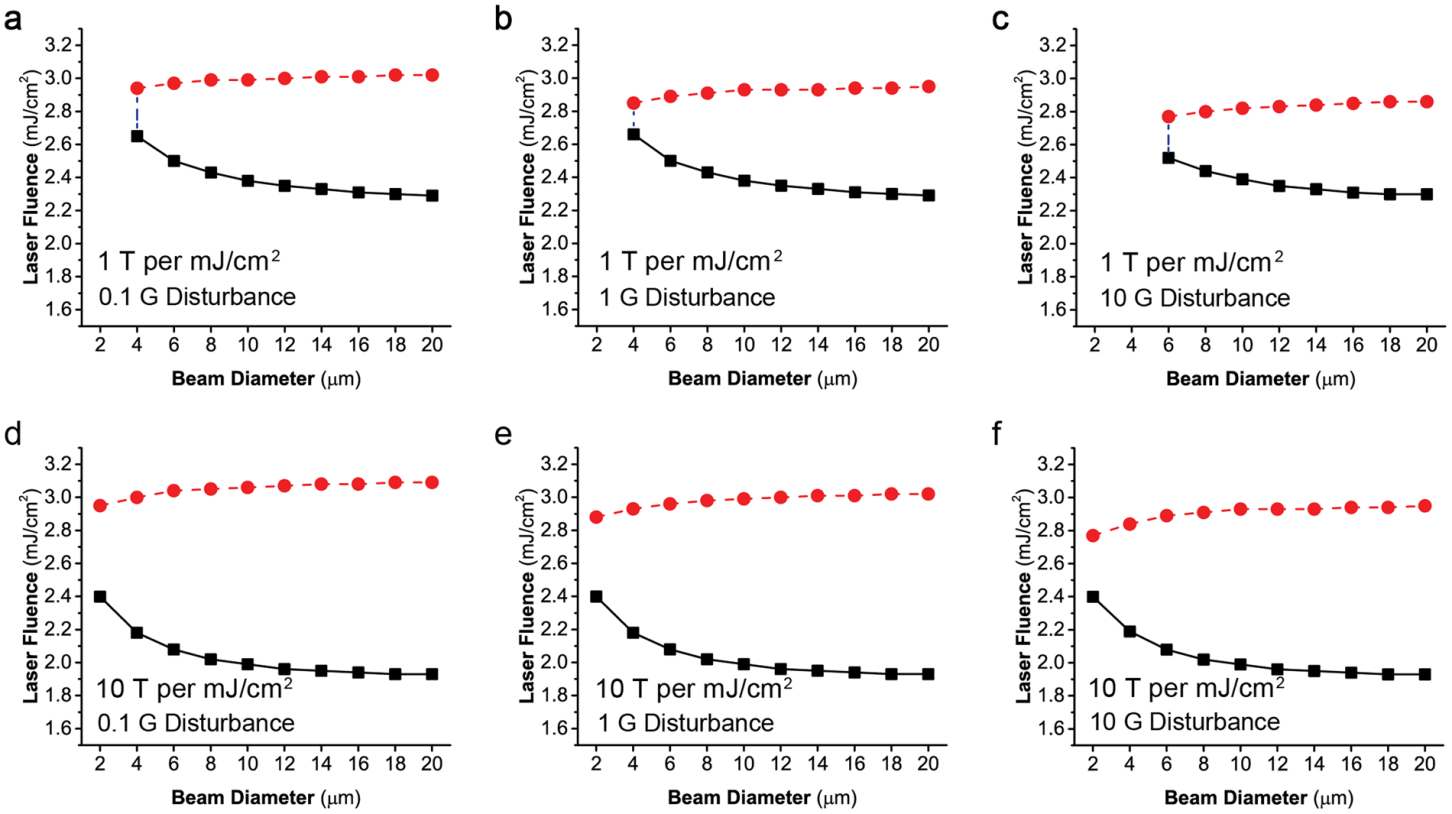
Phase diagrams of AOS under different magnetic disturbance levels. These curves are calculated using the analytical equation of estimated *B*_*i*_ at the center point of the laser beam. Two magneto-optical susceptibilities are used. (**a**,**b**) show the 1 T per mJ/cm^2^ cases. (**d**–**f**) show the 10 T per mJ/cm^2^ cases. The worst scenarios are considered here with constant disturbance levels (0.1–10 G) in the directions of narrowing the switched regions.

The analytical expression of the induced magnetic field allows to simplify the simulation and construct a scalar model to calculate the thresholds of AOS at the center of the laser beam (see Method section). Figure 7a–f shows the obtained the deterministic switching windows calculated using this scalar model under different levels of magnetic disturbance. The worst scenario is considered here with constant disturbance level (0.1–10 G) in the direction of narrowing the switched region. At a disturbance level of a few Gauss, the obtained thresholds can well reproduce the results in Fig. 5. The lower thresholds are nearly unaffected by disturbances up to 10 Gauss because the induced magnetic field is significantly stronger than the disturbances within the deterministic switching timescale (a few hundred fs). The upper thresholds are noticeably shifted downwards by the magnetic disturbances due to the increase of thermal settling time. Here, the thermal settling time means the time needed for the electron temperature to cool down below the Curie temperature. The increase of thermal settling time with large laser fluence is caused by the large heat capacity of the lattice. Near the upper thresholds, the lattice temperature is noticeably above the Curie temperature, and the thermal settling time increases to a few ps. So the upper thresholds are more sensitive to the disturbance. When the thresholds of AOS and demagnetization merge together with the increase of disturbance level, the deterministic switching cannot happen anymore. This can be seen in the 1 T per mJ/cm^2^ case in Fig. 7a–c. The cyan dashed line indicates the start point of deterministic switching on the beam diameter axis. Because of the large inverse Faraday effect, the absence of deterministic switching doesn’t happen in the 10 T per mJ/cm^2^ case, even though the deterministic region narrows with the increase of disturbance level.

### AOS near the edge of laser beams

**Figure 8:**
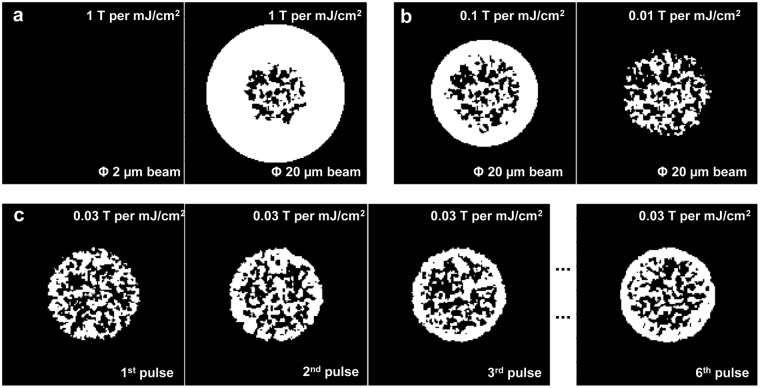
Cases of AOS at off-center locations at a laser fluence of 3.2 mJ/cm^2^, which can heat up the electrons to well above the Curie temperature at the center of the laser beam. (**a**) When IFE is strong enough (at magneto-optical susceptibility of 1 T per mJ/cm^2^), no AOS occurs for single pulse at a 2-µm laser beam size. For a single laser pulse of 20 µm size, the beam center is demagnetized while the edge of the laser beam becomes deterministically switched. (**b**) As the magneto-optical susceptibility reduces to 0.1 T per mJ/cm^2^, the edge-flip phenomena may still occur near beam edge but become weaker. And when it further reduced to the order of 0.01 T per mJ/cm^2^, no obvious AOS can be observed within the laser affected zone. (**c**) When the magneto-optical susceptibility is 0.03 T per mJ/cm^2^, the edge-flip phenomena can still occur when the material irradiated by multiple pulses. The side lengths for plot regions are all 15 µm.

AOS may also occur at a distance away from the beam center or near the beam edge which has been commonly observed in experiments^25,30,37^. In the ferromagnetic system, this has been demonstrated using multiple pulses, which shows the edge of the laser irradiated area is more likely to be deterministically flipped than the center^30,58^. This may occur when the laser fluence at the beam center starts to cause thermal demagnetization while the off-center locations instead experience favorable thermal and magnetic excitations and become deterministically switched. Especially when the laser beam is large, the laser induced B field near the edge of laser beam may last for a much longer time because of the accumulation of magnetic flux (*Φ*_*B*_) (Fig. S5 in Supplementary materials). Figure 8a shows the final magnetic state under the impacts of 2-µm and 20-µm laser beams with the same IFE amplitude (at magneto-optical susceptibility of 1 T per mJ/cm^2^). The laser pulse fluence is set to be 3.2 mJ/cm^2^ for all cases shown in Fig. 8. At the 2-µm laser beam size, there are no AOS because of the rapid decay of the induced magnetic field over the whole laser affected area. For the 20-µm beam size, the beam center is still demagnetized due to the highly-elevated electron temperature but the material near the edge of the laser beam becomes deterministically switched. Near this edge region, the material temperature is close to the Curie temperature meanwhile the induced magnetic field is prolonged and trigger the AOS. Similar phenomena also happen to the film with random initial magnetization (Fig. S6 in Supplementary materials). As the magneto-optical susceptibility reduces to 0.1 T per mJ/cm^2^, the edge-dominated-flip phenomena under 20-µm laser beams still exist but become noticeably weaker. When it further reduces to the order of 0.01 T per mJ/cm^2^, no obvious AOS can be observed within the laser affected zone for a single laser pulse irradiation. However, the edge-flip phenomena can still occur after repeated irradiations of multiple laser pulses. Figure 8c shows the evolution of the magnetization map under the impact of multiple laser pulses. The magneto-optical susceptibility is set to be 0.03 T per mJ/cm^2^ which is below the edge-flip threshold for single laser pulse. After the first pulse, no edge flip can be observed and the irradiated region becomes demagnetized. After the second and third laser pulses deposited onto the sample, the edge flip gradually shows up and becomes stabilized after more pulses. As the magneto-optical susceptibility further reduces, the require number of the laser pulses also grows accordingly. Here, we include a random noise with the root mean square level of ~6 Gauss and neglect the effect of M field on the induced B field. We also assume the pulse repetition rate is low enough to neglect the heat accumulations caused by multiple laser pulses.

## Conclusion

In this study, we numerically study the ultrafast deterministic AOS process for ferromagnetic material under a single laser pulse taking into account the realistic experimental conditions. We confirmed the dependence of AOS on the helicity of the single laser pulse and the existence and role of a prolonged B field which is induced by optothermal and optomagnetic couplings. Furthermore, the time sequence of the flipping process is studied based on four quantities (electron and lattice temperatures, material magnetization and induced magnetic flux density). We provided a fully coupled model, which is the first attempt to the best of our knowledge, to completely interpret the induced B field, including the magnitude and duration. The result predicts the possibility of ferromagnetic AOS under single laser pulse. We also studied the AOS process by varying the laser fluences and reproduced the AOS threshold of laser fluence which is similar to the ferrimagnetic experiment. The impacts of the laser fluence and beam diameter on the prolonged induced B field are studied systematically. The impacts of the magneto-optical susceptibility are also discussed. Based on these results, we provided a scalar model to predict the AOS process based on an analytical form of the prolonged induced B field.

For ferromagnetic material, the AOS was demonstrated in CoPt multilayer film using multiple pulses^30^ and the multiple pulse AOS and the edge-dominated-flip phenomena are also simulated using our model. It successfully reproduces the experimentally observed phenomena. Our result may be helpful to find a suitable material to demonstrate the single pulse AOS. To provide a physical picture in general, we also neglect the temperature dependence of thermal and optical properties except the heat capacity of the electrons. It is also worth mentioning that the physical origin of the gigantic magneto-optical susceptibility of AOS materials is still unclear and we employed an empirical value in the range suggested by other experimental and theoretic studies.

In summary, we numerically reproduced ferromagnetic AOS process and provide additional physical insights which are not conveniently available through experimental studies. We confirmed the existence and the mechanism of the prolonged induced B field. Our finding can help to optimize the performance of the AOS by constructing better AOS materials and tailoring the experimental parameters. For example, a magnetic material with high electrical conductivity will help to further prolong the induced B field and therefore help the deterministic switching.

## Methods

In our numerical study, we considered the underlying physics of optothermal and optomagnetic couplings by their thermodynamical natures. The in-phase and collective components of the optical responses can be considered using harmonic Maxwell’s equations which provide the driving terms for the optothermal and optomagnetic responses^59,60^. On the optothermal part, the optical energy dissipated from the in-phase and collective light-matter interactions is considered to be de-phased as Joule heating to generate hot carriers in the thermal transport processes. A two-temperature model is used to study the transient response of the electron and lattice systems under the sub-picosecond laser irradiation^43,61^. On the optomagnetic part, we use a classical approach in plasma science^55^ to calculate inverse Faraday effect using the optical field. The estimated amplitude of inverse Faraday effect is typically in the 0.1 to 30 T range in AOS^24,32,34,57,62,63^. A macroscopic Fockker-Planck and Landau-Lifshitz-Bloch (LLB) model is used to describe the transient response of the magnetization under finite temperature^46^. It is worthwhile to point out that the inverse Faraday effect in metals under femtosecond laser irradiation is still not fully understood yet. As alternatives, others proposed to explain this ultrafast optomagnetic response using magnetic circular dichroism^28,35–37^, the transfer of angular momentum^38,39^, the formation of a transient ferromagnetic state, and laser-induced superdiffusive spin currents and etc^10,35,38–41^. Meanwhile, material temperatures also affect the magnetic responses of the material considered through the material’s spin dynamics under finite temperature. This numerical scheme simplifies the numerical complexity of the problem and still preserves the numerical accuracy at the time scale of our interests. Similar numerical schemes have been validated to quantitatively study the ultrafast optothermal responses at different size scales under strong non-equilibrium and nonlocalities^64,65^. More details about optothermal and optomagnetic couplings can be found in Supplementary materials.

At the center of the laser beam, the AOS process can be estimated by a system of scalar equations including 1) two temperature model for material’s thermal response, 2) LLB model for material’s magnetization response, 3) the analytical expression of *B*_*i*_ for the magnetic excitation. The induced B field (*B*_*i*_) has the approximate analytical form as *B*_*i*_ = *B*_0_ + *f*(*t*)⊗*g*(*t*), where *B*_*0*_, *f*(*t*) and *g*(*t*) are the background B field, the excitation magnetic pulse and the relaxation function respectively. Their forms only explicitly depend on laser pulse and material parameters. Derivation for the analytical form of *B*_*i*_ can be found in Supplementary materials.

## Electronic supplementary material

Supplementary Information

Animation of magnetization field evolution under an RCP 2.5-mJ/cm2 laser pulse excitation (RCP.gif)

Animation of magnetization field evolution under a LINIEAR 2.5-mJ/cm2 laser pulse excitation (LIN.gif).

Animation of magnetization field evolution under an LCP 2.5-mJ/cm2 laser pulse excitation (LCP.gif).
