# Supplementary material for: Prediction of Deterministic All-Optical Switching of Ferromagnetic Thin Film by Ultrafast Optothermal and Optomagnetic Couplings
Source: Sci Rep. 2017 Oct 18;7:13513. doi: 10.1038/s41598-017-13568-w (PMC5647377; doi:10.1038/s41598-017-13568-w)
Supplement: Supplementary file 1 — Supplementary Information [file 41598_2017_13568_MOESM1_ESM.pdf]

Supplementary Information for

Prediction of Deterministic All-Optical Switching of Ferromagnetic Thin Film by Ultrafast Optothermal and Optomagnetic Couplings

Zhidong Du[1], Chen Chen[1], Feng Cheng[2], Yongmin Liu[2,*], Liang Pan[1,*]

[1] School of Mechanical Engineering and Birck Nanotechnology Center, Purdue University, West Lafayette, Indiana 47907, USA.

[2] Department of Mechanical and Industrial Engineering, Northeastern University, Boston, Massachusetts 02115, USA

** Correspondence and requests for materials should be addressed to Y.L. (email: y.liu@neu.edu) or L.P. (email: liangpan@purdue.edu).*

<u>List of Multimedia Files</u>

1. Animation of magnetization field evolution under an RCP 2.5-mJ/cm^2 laser pulse excitation (RCP.gif).
2. Animation of magnetization field evolution under a LINIEAR 2.5-mJ/cm^2 laser pulse excitation (LIN.gif).
3. Animation of magnetization field evolution under an LCP 2.5-mJ/cm^2 laser pulse excitation (LCP.gif).

Supplementary Figures

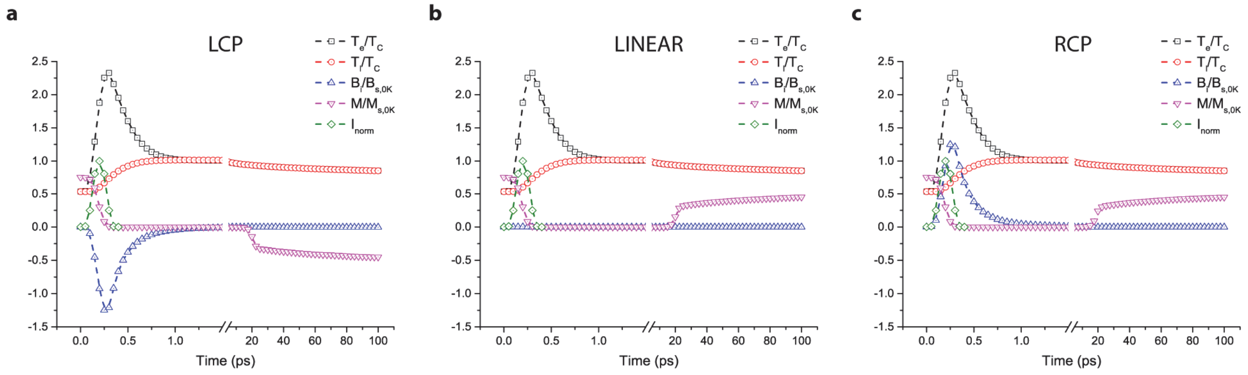

Figure S1. Time evolution of the four important quantities under different laser helicities: electron temperature (*T_e*), lattice temperature (*T_l*), induced magnetic flux density *B_i* and magnetization *M*, when an M^+ magnetic medium is irradiated by (a) a single LCP (σ^-) pulse, (b) a single linearly-polarized (L) pulse, and (c) a single RCP (σ^+) pulse. Under 2.6 mJ/cm^2 lase pulse, only the LCP helicity pulse can flip the magnetization.

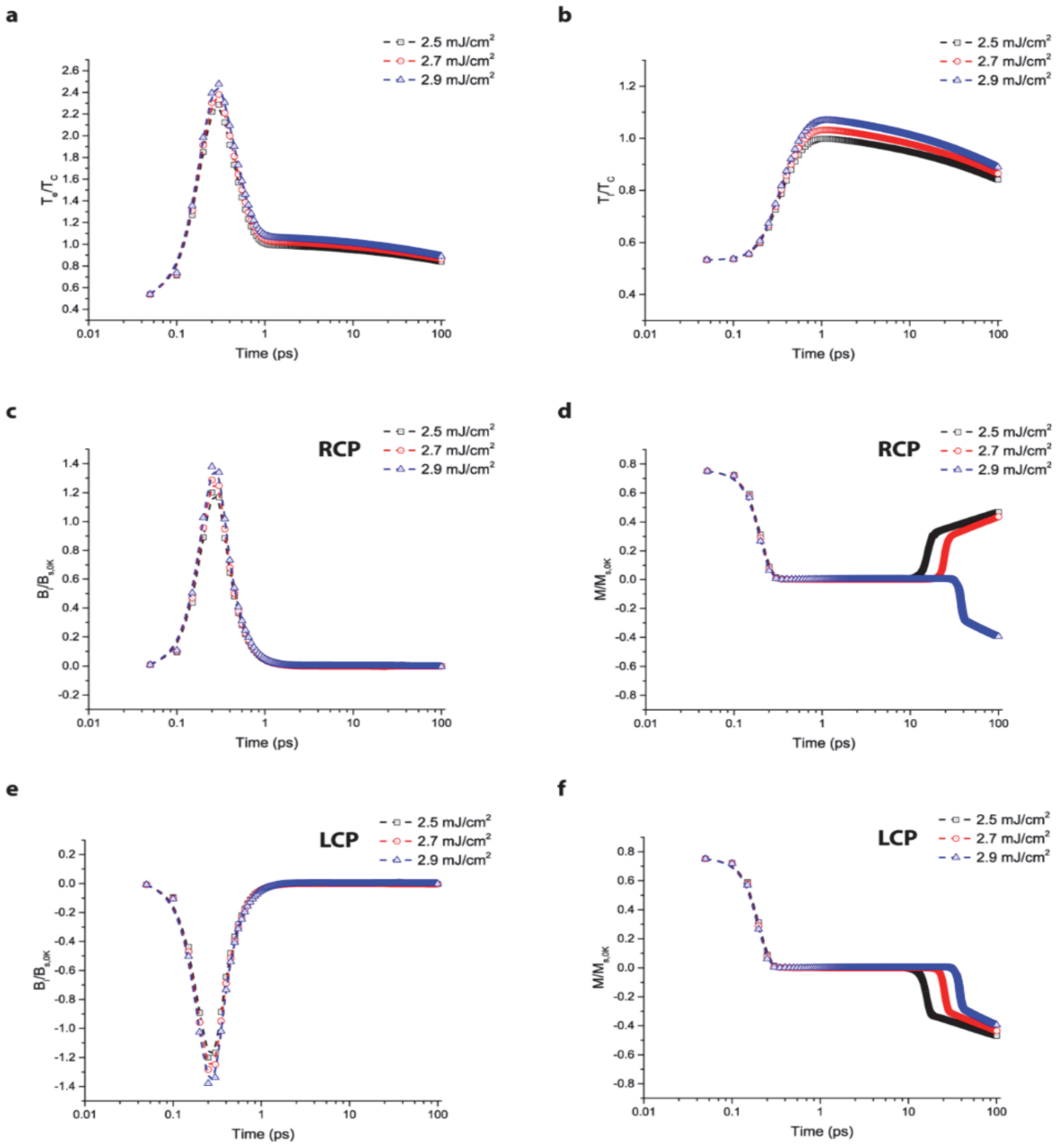

Figure S2. Time evolutions of T_e, T_l, B_i and M under RCP (σ^+) and LCP (σ^-) laser pulses with different fluences. Demagnetization occurs at the laser fluence where the final magnetization state is not determined by the helicity of the laser pulse. It's assumed that the temperatures of electron and lattice systems don't depend on the laser helicity. The laser beam diameter is 10 μm. Figure S2 a-b show the temperatures under the laser fluence ranging from 2.5 to 2.9 mJ/cm^2. Figure S2 c-f show the $\boldsymbol{B_i}$ and $\boldsymbol{M}$ fields under RCP and LCP laser pulses, respectively. By comparing Fig. S2 d and f, it can be seen that no matter what helicities the laser pulse has, the M field can be flipped under large laser fluence (2.9 mJ/cm^2). This can be seen as criterion of demagnetization.

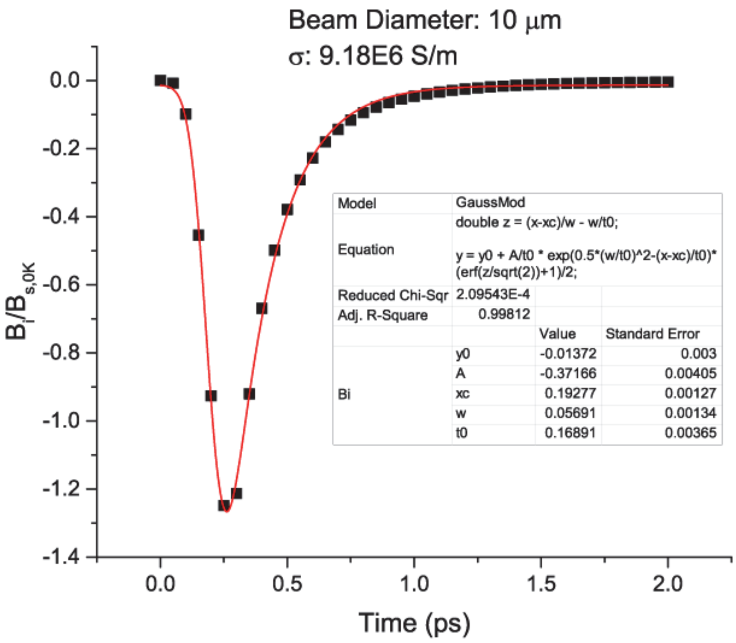

Figure S3. Fitting of B_i by the analytical expression. If the inverse Faraday effect is large compared with the M field, B_i can be approximated by an analytical expression of convolution of laser pulse and exponential decay. Figure S3 shows that the fitting is very good.

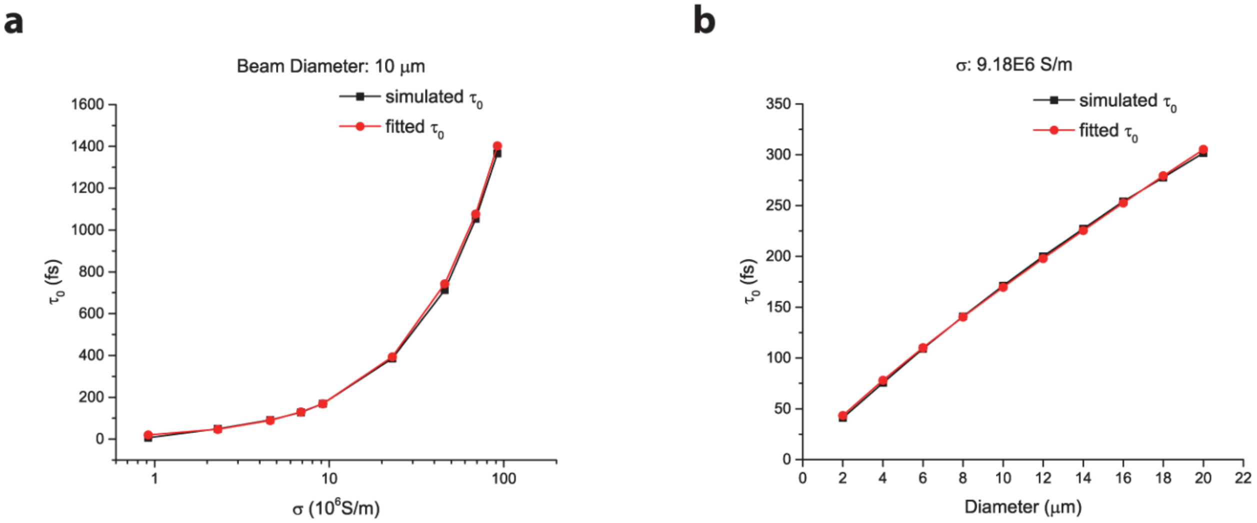

Figure S4. Fitting of τ_0 by the empirical expression. The decay time constant can be fitted by an empirical expression depending on the material electrical conductivity σ and the laser beam diameter D. Figure S4 shows that the empirical expression is accurate.

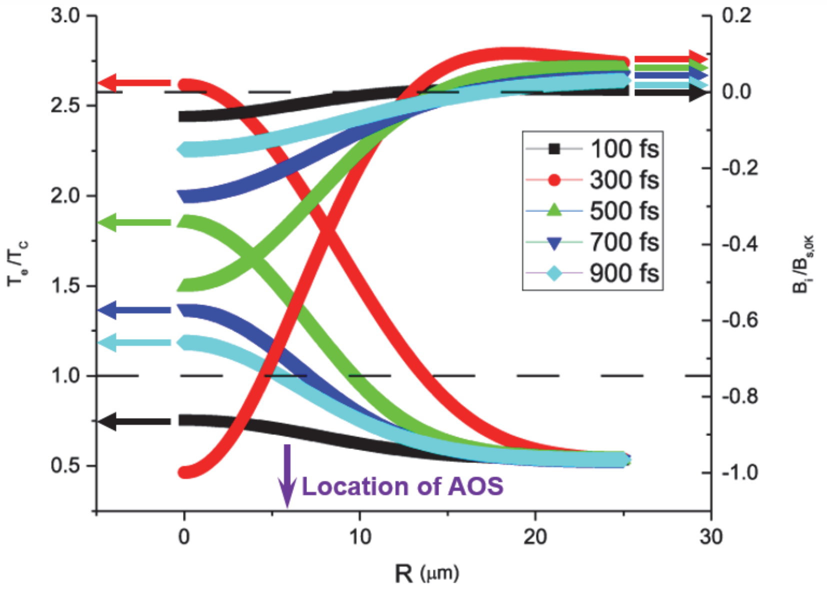

Figure S5. T_e and B_i evolution over time. The edge of the laser beam has B_i field larger than noise level during T_e above the Curie temperature. This favors the edge-dominated flipping. The center temperature T_e is above the Curie temperature for too long time and result in random magnetization.

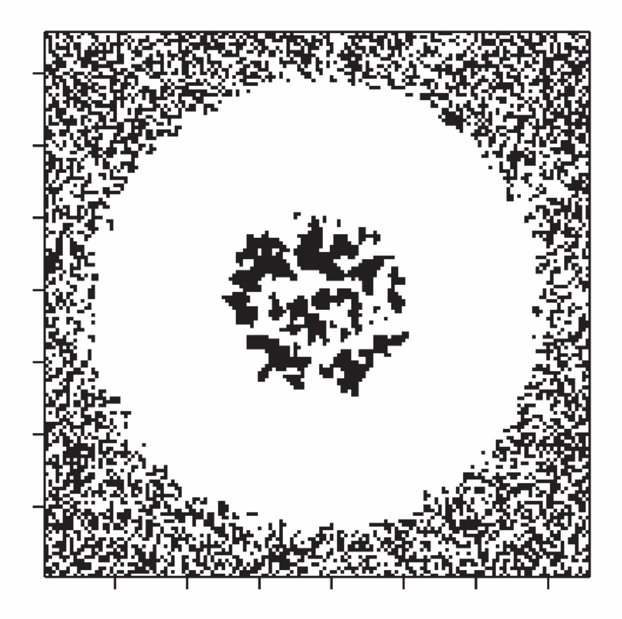

Figure S6. Edge-dominated flip with random initial magnetization. Edge can be flipped by 20-μm laser beam pulse with random initial magnetization. The side length of the plotted area is 15μm.

Supplementary Methods

Two-Temperature Model

The transient response of the electron and lattice temperatures under the sub-picosecond laser irradiation are calculated as below[S1, S2].

$$\frac{\partial T_e}{\partial t} = \alpha_e \nabla^2 T_e - G_{el}(T_e - T_l) + \dot{Q}_{laser} \tag{1}$$

$$\frac{\partial T_l}{\partial t} = \alpha_l \nabla^2 T_l + G_{el}(T_e - T_l) \tag{2}$$

where $\alpha_{e,l} = \left(k/\rho C_p \right)_{e,l}$ is the thermal diffusivity, $\dot{Q}_{laser}$ is volumetric heating from the laser irradiation and G_{el} is the thermal coupling constant between electron and lattice systems. The heat capacity of electron system is modeled as

$$C_{p,e} = \gamma_e T_e \tag{3}$$

The electron system absorbs the optical heating and its temperature peaks in a few hundreds of femtoseconds. Simultaneously electron system transfers its thermal energy to lattice system via the electron-lattice interaction. The electron-lattice system approximately reaches their local equilibrium at about 1 picosecond. Eventually, the thermal energy diffuses into its neighbors.

Macroscopic Fockker-Planck and Landau-Lifshitz-Bloch (LLB) model

The macroscopic LLB model is used to describe the transient response of the magnetization as follow

$$\dot{\boldsymbol{M}} = \gamma[\boldsymbol{M} \times \boldsymbol{B}_{eff}] - L_1 \frac{\left(\boldsymbol{M} \cdot \boldsymbol{B}_{eff}\right)\boldsymbol{M}}{M^2} + L_2 \frac{\boldsymbol{M} \times \left(\boldsymbol{M} \times \boldsymbol{B}_{eff}\right)}{M^2}, \tag{4}$$

where γ is the gyromagnetic ratio. L_1 and L_2 are the longitudinal and transverse kinetic coefficients.

$$L_1 = \gamma M_{s,0K} \lambda \frac{2T_e}{3T_C} \tag{5}$$

$$L_2 = \begin{cases} \gamma M_{s,0K} \lambda (1 - \frac{T_e}{3T_C}), T_e < T_C \\ \gamma M_{s,0K} \lambda \frac{2T_e}{3T_C}, T_e \geq T_C \end{cases} \tag{6}$$

The λ charaterizes the coupling strength between the spin and the thermal bath. Below the Curie temperature, Gilbert damping parameters $\alpha_{1,2}$ can be expressed as,

$$\alpha_1 = \frac{2\lambda}{m_e} \frac{T_e}{3T_C} \tag{7}$$

$$\alpha_2 = \frac{\lambda}{m_e} \left(1 - \frac{T_e}{3T_C} \right) \tag{8}$$

where m_e is the equilibrium spin polarization at finite temperature.

The $\boldsymbol{B}_{eff}$ has the form of

$$\boldsymbol{B}_{eff} = \boldsymbol{B}_i + \boldsymbol{B}_{ani} + \mu_0 (\frac{1}{q_d^2} \Delta \boldsymbol{M} + \boldsymbol{H}_J), \tag{9}$$

$$\boldsymbol{H}_J = \begin{cases} \frac{1}{2\chi_{\parallel}} \left(1 - \frac{M^2}{M_s^2} \right) \boldsymbol{M}, T_e < T_C \\ -\frac{1}{\chi_{\parallel}} \left(1 + \frac{3}{5} \frac{T_e}{T - T_C} \frac{M^2}{M_{s,0K}^2} \right) \boldsymbol{M}, T_e \geq T_C \end{cases} \tag{10}$$

where $\boldsymbol{B}_i$ is the induced magnetic flux density, $\boldsymbol{B}_{ani}$ is the anisotropy field, $\frac{1}{q_d^2} \Delta \boldsymbol{M}$ is the magnetic domain interaction field, $\boldsymbol{H}_J$ is the internal exchange field and $\chi_{\parallel}$ is the longitudinal susceptibility which is defined as $\chi_{\parallel} = \left(\frac{dM_{\parallel}}{dH_{ext,\parallel}} \right)_{H_{ext,\parallel} \to 0}$. For the spin dynamics under finite temperature, this macroscopic Fockker-Planck and Landau-Lifshitz-Bloch equations can capture and reproducible the collective evolution of magnetization while other spin dynamics can be numerically treated as general magnetic disturbances[S3].

In supplementary equation (4), L_1, L_2, and $\boldsymbol{H}_J$ depend on the electron temperature T_e and they will reflect the phase change when the electron temperature is above the Curie temperature T_C. The spin precession plays a minor role in the AOS process[S4] therefore we neglect the first and third term of supplementary equation (4). The anisotropy field is set to be zero in our analysis.

Inverse Faraday Effect

The classical approach in plasma science calculates inverse Faraday effect[S5] as

$$\boldsymbol{H}_{IFE} = \alpha[\boldsymbol{E} \times \boldsymbol{E}^*], \tag{11}$$

where α is the magneto-optical susceptibility. The inverse Faraday effect serves as a transient source term $\boldsymbol{j}_{IFE}=\nabla\times\boldsymbol{H}_{IFE}$ during the magnetic field calculation. In this treatment, the $\partial\boldsymbol{E}/\partial t$ term is neglected which is an acceptable approximation in AOS process.

Approximate expression of the induced magnetic field $\boldsymbol{B_i}$

The induced B field only explicitly depends on laser and material parameters and has the approximate analytical form as

$$B_i(t) = B_0 + \frac{\alpha c \mu_0^2 I_{laser}}{\tau_0\sqrt{8\ln 2}} \exp\left[\frac{1}{2}\left(\frac{w}{\tau_0}\right)^2 - \frac{t-t_0}{\tau_0}\right](erf(\frac{t-t_0}{\sqrt{2}w} - \frac{w}{\sqrt{2}\tau_0}) + 1). \tag{12}$$

This B_i is calculated from $B_i = B_0 + f(t)\otimes g(t)$, where $f(t) = \frac{F_{IFE}}{w\sqrt{2\pi}}\exp\left[-\frac{(t-t_0)^2}{2w^2}\right]$, $g(t) = \frac{1}{\tau_0}\exp\left(-\frac{t}{\tau_0}\right)\cdot u_0(t)$ and $u_0(t)$ is the unit step function. B_0 is the background B field. τ_0 is the decay time constant and t_0 is the time of the laser peak. The treatment of convolution is accurate because Maxwell's equations are linear with respect to the loop current $\boldsymbol{j}$ in the absence of material nonlinearity or variations in M field. We approximate the relaxation function $g(t)$ as an exponential decay. Although the realistic decay time constant in $g(t)$ also varies with time, this approximate can still effectively capture the B_i relaxation at the initial stage of the AOS (Fig. S3). The magnetic disturbances can be modeled by adding more terms to the analytical expression of the induced magnetic field.

The laser duration is represented by w. F_{IFE} is related to the laser fluence I_{laser} by

$$F_{IFE} = \sqrt{\frac{\pi}{\ln 2}}\alpha c \mu_0^2 I_{laser}. \tag{13}$$

The accuracy of the analytical expression is validated by the curve fitting shown in Fig. S3.

The decay time constant τ_0 (in fs) is found to be a function of electrical conductivity σ (in 10^6S/m) and the laser beam diameter D (in μm) as

$$\tau_0 = C\sigma^m D^n \tag{14}$$

For the 10-nm thick ferromagnetic material ($\sigma = 9.18\times10^6$ S/m) used in this study, *C*, *m*, and *n* are fitted from the numerical results as 3.16, 0.917 and 0.847 respectively. This correlation is accurate to the level of 6% for a range of σ (2.30-91.8 10^6S/m) and *D* (2-20 μm) which is shown in Fig. S4.

Scalar Model

The scalar model is derived to simplify and reduce the simulation time. The electron temperature is calculated by the two-temperature model. Only the z component of the M field is considered and the analytical expression of B_i is used.

$$\dot{M} = -L_1(B_i + B_{disturbance} + \mu_0 H_J) \tag{15}$$

Figure 6 in the main text is generated by solving supplementary equations (1), (2), (12) and (15).

Numerical implementation

The detailed numerical studies in this work are carried out using commercial multi-physics software COMSOL. We used Heat Transfer in Solids, Magnetic Fields modules to capture thermal and magnetic responses respectively. The in-house module for material magnetization is constructed by using the generic mathematics model for the macroscopic LLB equation. The axial symmetric geometry is modeled under the cylindrical coordinates with minimum grid size about 3 nm.

The virtual ferromagnetic material parameters for the numerical model are list in Table S1.

Table S1

$M_{s,0K}$	800000 A/m
λ	0.03
T_C	550 K
α	2.13×10^{-11} A·m/V^2
γ_e	665 J/(m^{3}K^2)
$\alpha_e = k_e / \rho_e \gamma_e T_e$	0.0925/T_e m^2/s
α_l	1.67×10^{-6} m^2/s
α_{glass}	7.83×10^{-7} m^2/s
G_{el}	2.6×10^{18} W/(m^{3}K)
σ	9.18×10^{6} S/m

References

S1. Guo L, Xu X. Ultrafast spectroscopy of electron-phonon coupling in gold. Journal of Heat Transfer 136, 122401-122401 (2014).

S2. Qiu TQ, Tien CL. Heat-transfer mechanisms during short-pulse laser-heating of metals. Journal of Heat Transfer-Transactions of the ASME 115, 835-841 (1993).

S3. Garanin DA. Fokker-Planck and Landau-Lifshitz-Bloch equations for classical ferromagnets. Physical Review B 55, 3050-3057 (1997).

S4. Vahaplar K, et al. Ultrafast path for optical magnetization reversal via a strongly nonequilibrium state. Phys Rev Lett 103, 117201 (2009).

S5. Hertel R. Theory of the inverse Faraday effect in metals. Journal of Magnetism and Magnetic Materials 303, L1-L4 (2006).
