# Supplementary figures and images for: Prediction of Deterministic All-Optical Switching of Ferromagnetic Thin Film by Ultrafast Optothermal and Optomagnetic Couplings
Source: Sci Rep. 2017 Oct 18;7:13513. doi: 10.1038/s41598-017-13568-w (PMC5647377; doi:10.1038/s41598-017-13568-w)

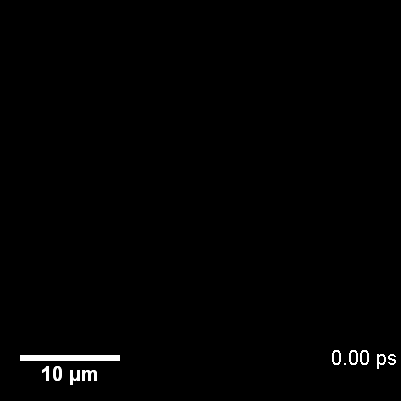

Supplement: Supplementary file 2 — Animation of magnetization field evolution under an RCP 2.5-mJ/cm2 laser pulse excitation (RCP.gif) [file 41598_2017_13568_MOESM2_ESM.gif]

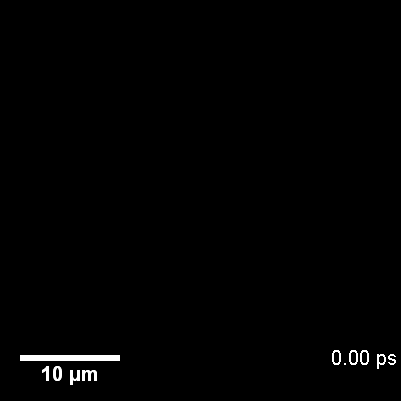

Supplement: Supplementary file 3 — Animation of magnetization field evolution under a LINIEAR 2.5-mJ/cm2 laser pulse excitation (LIN.gif). [file 41598_2017_13568_MOESM3_ESM.gif]

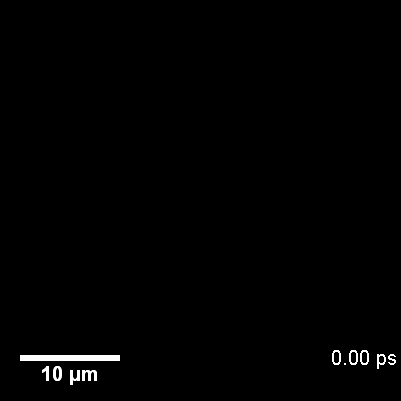

Supplement: Supplementary file 4 — Animation of magnetization field evolution under an LCP 2.5-mJ/cm2 laser pulse excitation (LCP.gif). [file 41598_2017_13568_MOESM4_ESM.gif]
